# The Current Status and Future Perspectives of Chimeric Antigen Receptor-Engineered T Cell Therapy for the Management of Patients with Endometrial Cancer

**DOI:** 10.3390/cimb45040220

**Published:** 2023-04-12

**Authors:** Ji-Young Choi, Tae-Jin Kim

**Affiliations:** 1 Department of Gynecology and Infertility Medicine, CHA University Ilsan Medical Center, Goyang 1205, Republic of Korea; jychoi12@chamc.co.kr; 2 Department of Urology, CHA University Ilsan Medical Center, CHA University School of Medicine, Goyang 1205, Republic of Korea

**Keywords:** gynecologic malignancies, CAR-T, immunotherapy, endometrial cancer, T cells

## Abstract

Endometrial cancer (EC) is a gynecological neoplasm that is increasing in occurrence and mortality rates. Although endometrial cancer in the early stages shows a relatively favorable prognosis, there is an increase in cancer-related mortality rates in the advanced or recurrent endometrial carcinoma population and patients in the metastatic setting. This discrepancy has presented an opportunity for research and development of target therapies in this population. After obtaining promising results with hematologic cancers, chimeric antigen receptor (CAR)-T cell immunotherapy is gaining acceptance as a treatment for solid neoplasms. This treatment platform allows T cells to express tumor-specific CARs on the cell surface, which are administered to the patient to treat neoplastic cells. Given that CAR-T cell therapy has shown potential and clinical benefit compared to other T cell treatment platforms, additional research is required to overcome physiological limitations such as CAR-T cell depletion, immunosuppressive tumor microenvironment, and the lack of specific target molecules. Different approaches and development are ongoing to overcome these complications. This review examines CAR-T cell therapy’s current use for endometrial carcinomas. We also discuss the significant adverse effects and limitations of this immunotherapeutic approach. Finally, we consolidate signal-seeking early-phase clinical trials and advancements that have shown promising results, leading to the approval of new immunotherapeutic agents for the disease.

## 1. Introduction

Due to an increase in life expectancy and higher obesity rates, numerous reports have shown that endometrial cancer (EC) is rapidly growing in incidence, becoming the most common gynecologic cancer in the United States. In 2022, it was estimated by the American Cancer Society that there would be over 65,950 new cases of EC diagnosed and 12,550 cases of mortality [1]. Although recent developments in molecular biology have furthered our understanding of the underlying pathologic mechanisms of EC, many aspects of treatment modalities, such as the selection of end-stage patients for the administration of adjuvant chemotherapy or radiotherapy, remain controversial.

Despite an excellent prognosis in patients diagnosed in the early stages of EC, patients with metastatic or recurrent disease have limited treatment options after their initial response to first-line chemotherapy. The standard treatment for patients with advanced disease or a high risk of recurrence is adjuvant radiotherapy (RT). A combination regimen of carboplatin/paclitaxel systemic chemotherapy and RT can benefit this patient population. As for patients with recurrent or metastatic cancer, conventional combination therapy with or without RT is considered appropriate. However, the number of therapeutic options decreases for patients diagnosed with advanced disease after unsuccessful standard treatments [2]. This warrants studies and investigation of alternative modalities and treatment regimens for this patient population.

Research and technological advances are ongoing to further develop treatment approaches to reduce mortality and recurrence of tumors and improve the survival rate of patients. Immunotherapy has been noteworthy as a novel method to overcome unresponsive recurrent malignancies [3,4,5]. Immune checkpoint inhibitor (ICI) therapy has shown potential as an effective treatment modality. In 2017, pembrolizumab was approved by the Food and Drug Administration (FDA) for the treatment of EC patients with mismatch repair deficiency (dMMR) or high microsatellite instability (MSI-H) tumors. Following the positive results of pembrolizumab, further studies on other ICI agents for this specific population ensued, and after showing therapeutic benefit and an acceptable safety profile, dostarlimab was granted accelerated approval from the FDA for the treatment of dMMR recurrent or advanced EC in 2021 [6]. The clinical efficacy of ICI immunotherapy has set a trend to investigate the role of immune checkpoint blockade as a first-line treatment option for EC patients in the refractory setting. However, further clinical research is required for possible treatment strategies of future EC patients who will experience recurrence after ICI immunotherapy.

After promising results in treating hematological cancers, chimeric antigen receptor (CAR)-T cell therapy has shown promise as an alternative treatment for EC patients who have failed chemotherapy [6]. Adoptive cellular therapy (ACT) is one of these promising new methods. In this therapy, T cells taken from the peripheral circulation are collected via leukapheresis and genetically modified ex vivo before reinfusion. ACT has been shown to have therapeutic benefits [7].

Several ACT therapies, such as tumor infiltration lymphocytes (TILs), engineered T cell receptors (TCR), and CAR-T cell therapy, have been administered as treatment modalities, and the therapeutic benefits are being evaluated in various clinical settings and studies [8]. However, further research related to CAR-T cell therapy for EC patients is warranted. In a preclinical study by Rodriguez-Garcia et al., looking at treatment outcomes of patients with ovarian and EC, anti-Mullerian inhibiting substance type II receptor (MISIIR) CAR-T cell therapy was evaluated [9]. MISIIR is classified as a part of the transforming growth factor-β receptor family. Various studies have shown that most gynecologic cancers, including EC, have high expression levels of this receptor, making it a prospective target for administering CAR-T cells [10,11,12].

This preclinical study showed that MISIIR-specific CAR-T cells demonstrated antigen-specific reactivity and eradicated MISIIR-overexpressing tumor cells. The study showed that CAR-T cell therapy specifically targeting MISIIR for treating ovarian cancer and other gynecologic cancers is possible. This article provides a comprehensive overview of CAR-T cell therapy for EC patients. It also reviews current and future considerations that will help to improve the therapeutic continuum for advanced and metastatic diseases.

## 2. Immunotherapy in the Treatment of Endometrial Cancer

### 2.1. The Interaction between the Female Endometrium and the Immune System

The immune environment in normal endometrium contains inflammatory cytokines, endometrial epithelial cells, and innate and adaptive immune cells [13]. Inflammatory cytokines play a significant role in recruiting migratory immune cells and regulating the endometrial immune system. Endometrial epithelial cells function as an anatomical barrier and by producing immune mediators. They also play a role in antigen presentation [14,15,16,17].

Immune surveillance and pathogen elimination in the endometrium are controlled by innate and adaptive immune cells [13]. The immune system and endometrial tissue have a complex relationship, and this process is meticulously controlled by the serum estradiol and progesterone levels that rise and fall in the normal menstrual cycle [13,16,18]. Macrophages and neutrophils attain their highest levels before menstruation, where they play crucial roles in endometrial disruption and immunologic protection.

In comparison, the adaptive immune system is activated by leukocyte aggregates comprising B cells, T cells, and macrophages, which increase in population during the proliferative period. Cytotoxic activities are reduced during the secretory period to create an optimal environment for conception [19,20,21]. Endometrial immunity is a dual-purpose system that influences physiological changes. It creates an immunosuppressive environment to prevent fetomaternal rejection while safeguarding the endometrium from pathogens and sexually transmitted diseases during menstruation [13].

### 2.2. Rationale of CAR-T Cell Immunotherapy for the Management of Endometrial Cancer

ACT is an effective treatment method in immunotherapy, and the manipulation and production of TILs is one of the most significant achievements in this therapeutic model. In early 2019, the Food and Drug Administration approved a breakthrough designation for the LN-145 TIL therapy to manage cervical cancer; this has prompted further interest in ACT for treating other gynecological neoplasms [22].

TILs may have high numbers of T cells, but these cells have an extremely low survival rate and life expectancy due to their fragile molecular properties and exposure to the unfavorable tumor environment (TME). Harvesting TILs and their lymphocytes necessitates invasive surgery. To solve some of these issues, genetically designed T cells are being developed, which include T cell receptor-modified T cells (TCR-Ts) and CAR-T cells. These newer cells can be genetically manipulated and modified to interact with specific tumor antigens or express cytokines that can protect cells from the harsh immunosuppressive milieu of the TME. These advancements further the clinical spectrum of immunotherapy.

Compared to TCR-Ts and TILs, CAR-T cells have molecular properties beneficial in the immunotherapy setting. CAR-T cells are not limited by the major histocompatibility complex (MHC) due to the immunological characteristics of their surface–antigen interaction. A benefit of CAR-T cell therapy is their molecular property, which is crucial in managing neoplasms with a low MHC expression and a resistance to TCR-Ts or TILs. Most TCRs have a low antigen affinity, leading to off-target adverse events, whereas CAR-T cells have a lower occurrence of these toxicities [23]. CAR-T cells also have T cells’ antigen binding capacity and cell lysis capabilities, making CAR-T cell immunotherapy a favorable option for treatment [24].

## 3. The Molecular Schematics of CAR-T Cells

### 3.1. Molecular Components and Structure of CAR-T Cells

CAR-T cells are genetically assembled MHC complex-independent molecules in which the effector part of the T lymphocytes recognizes and interacts with tumor-associated antigen (TAA)-expressing cells, resulting in an antitumor response. This cellular interaction occurs in a setting where the immunologic activity of antigen-presenting cells is not a prerequisite. This is a distinct characteristic compared to the necessary molecular-signaling cascade for unmodified T cell activation.

The primary role of the CAR molecule is recognizing target antigens and facilitating intracellular signaling between the transmembrane and intracellular regions. The intracellular costimulatory domains in the CAR are the important characteristic that differentiates each CAR-T cell generation. This variance in the costimulatory construct regulates the immunologic complexity, molecular phenotype, and capability of CAR-T cells; it is the primary factor that defines the immunotherapeutic scope of the second- or third-generation CAR constructs [23].

CAR molecules are composed of distinct components. The extracellular domain has a single-chain fragment variable (scFv) structure that attaches to the TAA, and its purpose is to identify the target antigen. The transmembrane domain is composed of the transmembrane region’s cluster of differentiation (CD) 3, CD 8, CD 28, or FcεRI. This enables the attachment of scFv to the T cell, while the transmembrane area is connected to the intracellular regions located in the intracytoplasmic domains of CD 8, CD 28, CD 137, and CD 3ζ. The intracellular zone includes the immune receptor tyrosine-based activation motif (ITAM) that is required for the signaling of T cell activation [25].

### 3.2. Molecular Generations of CAR-T Cells

**Figure 1:**
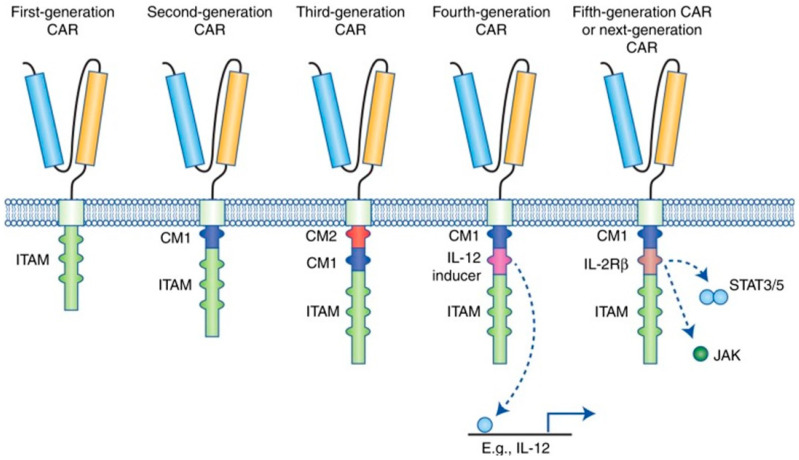
Molecular configuration of chimeric antigen receptor (CAR) generations. The first-generation CAR only contains immune receptor tyrosine-based activation motifs (ITAM) in the intracellular domain. Second-generation CARs incorporated one costimulatory molecule, and third-generation CARs combined a second costimulatory molecule. As the fourth-generation CAR, the TRUCK shares the molecular configuration of second-generation CAR with the addition of a constitutive cytokine inducer. The fifth-generation CAR shares the molecular construct of the second generation, with the additional intracellular domains of cytokine receptors such as the IL2R ß chain fragment [38]. CAR: chimeric antigen receptor, scFv: single-chain fragment variable, ITAM: immunoreceptor tyrosine-based activation motifs, TRUCK: T cell redirected for universal cytokine-mediated killing, IL2R ß: IL-12 receptor ß chain domain.

In the past three decades, there have been major advances in the development of CARs. With these advances, it is vital to acknowledge the new molecular designs and modifications in recent generations of this treatment modality. CARs are divided into five generations based on their molecular structure (Figure 1). These CARs will be discussed in further detail below.

#### 3.2.1. First Generation of CAR-T Cells

CAR-T cells of the first generation contain a single receptor divided into the scFv, transmembrane domain, and intracellular zone. This molecular structure made it difficult for CAR-T cells to maintain an adequate population of activated T lymphocytes in the circulatory system. This was due to the limited CAR-T cell ability to activate T cells without a costimulatory molecule [26,27,28].

#### 3.2.2. Second Generation of CAR-T Cells

Second-generation CARs were modified to insert an extra costimulatory protein such as CD 28, CD 27, CD 134, or CD 137 into the intracellular domain to overcome this obstacle. This modification allowed the CAR-T cells to deliver a secondary signal when encountering a tumor antigen. Preclinical and clinical studies showed that the presence of an extra costimulatory protein enhanced proliferation and cytotoxicity while improving response due to a longer persistence of active CAR-T cells in circulation [26].

#### 3.2.3. Third Generation of CAR-T Cells

Third-generation CAR-T cells had additional costimulatory molecules (i.e., CD 28, 4-1BB, and CD 3ζ) to increase the number of activated T cells in the blood circulation [29]. Although the third generation has shown promising results in certain types of cancer with acceptable safety profiles, there were no advancements in persistence, proliferation, or efficacy when compared to the second generation of CAR-T cells [29].

#### 3.2.4. Fourth Generation of CAR-T Cells

The cells in the fourth generation of the CAR-T cell family are the T cells redirected for universal cytokine-mediated killing (TRUCKs). In this model, a costimulatory domain and pro-inflammatory configuration, such as interleukin (IL)-12, were inserted to increase the effectiveness of circulating T cells [30]. The purpose of IL-12 is to lessen the immunosuppressive characteristics of the TME by activating the T helper 1-type cell cascade [31,32]. Amendments to the fourth-generation CARs are not limited to IL-12, and numerous molecular constructs have been fabricated or redesigned to be inserted into the TRUCK assembly. Other variations include cytokines such as IL-15 to increase T-memory stem cells and IL-18 [33,34]. IL-18 activates interferon-γ (IFN-γ) secretion, enhances the T cell immune response, and is responsible for natural killer (NK) cell and cytotoxic T cell activation [33]. Avanzi et al. showed that IL-18-secreting CAR-T cells displayed enhanced cellular expansion and persistence in murine studies of hematological and solid cancers [33]. One of the major setbacks of CAR-T cell therapy is due to the limited expansion and persistence of the aforementioned cells. To overcome this limitation, researchers constructed a modified IL-7 receptor by inserting cysteine into the transmembrane domain while replacing the natural extracellular domain of the receptor with the extracellular components of CD34, which was named C7R. The addition of C7R into CAR-T cells results in the increase in cell numbers and cell survival [35]. Knock-out genes (e.g., PD-1 or diacylglycerol kinase) and knock-in genes (e.g., the TCRα subunit constant gene or the chemokine C-X-C motif receptor [CXCR] 4) are molecular constructs added into the TRUCK cassette to augment CAR expression and increase antitumor effects [36,37]. Due to the complex and immunosuppressive nature of the TME, the directing and trafficking of CAR-T cells are essential for therapeutic purposes. Since the chemokine cascade has a role in cell migration during hematopoiesis during cellular development, utilizing this function to guide CAR-T cells to the target tumor is possible with the addition of chemokine receptors such as CXCR4. Moreover, controlled trafficking of CAR-T cells presents therapeutic advantages as it results in decreased dissemination of CAR-T cells and allows for a lower administered dosage to the patient [36,37]. In addition, to stimulate molecular expression while counteracting antigen escape, controlled and inducible systems (Syn/Notch) and antigen combinations of human epidermal growth factor receptor 2 (HER 2) + IL-13 receptor subunit α2 (IL-13Rα2) have been fabricated [24].

#### 3.2.5. Fifth Generation/Next Generation of CAR-T Cells

To overcome the limitations of the previous CAR-T cell generations, the fifth-generation CAR-T cell was constructed. The molecular design is similar to the second-generation CAR-T cell, the difference being the insertion of a truncated cytoplasmic IL-12 receptor ß chain domain (IL2R ß). The purpose of the additional intracellular domain was to bind signal transducer and activator of transcription 3 (STAT3). The activation of this receptor results in cell proliferation while minimizing terminal differentiation and increasing CAR persistence [38,39]. Figure 1 illustrates the molecular structure of the individual CAR-T cell generations.

### 3.3. Preparation and Administration of CAR-T Cell Therapy

Preparation of CAR-T cell therapy begins by acquiring lymphocytes from the peripheral circulation via leukapheresis, followed by apheresis without the additional step of adding granulocyte colony-stimulating factor (G-CSF) [40]. The T cells are genetically engineered to be infected with either a retroviral/lentiviral vector or a non-viral CAR vector in which genomic DNA is implanted. After molecular expansion and ex vivo purification, the refined CAR-T cells are reintroduced into the circulatory system of the patient, who had previously undergone lymphodepletion [40,41].

**Figure 2:**
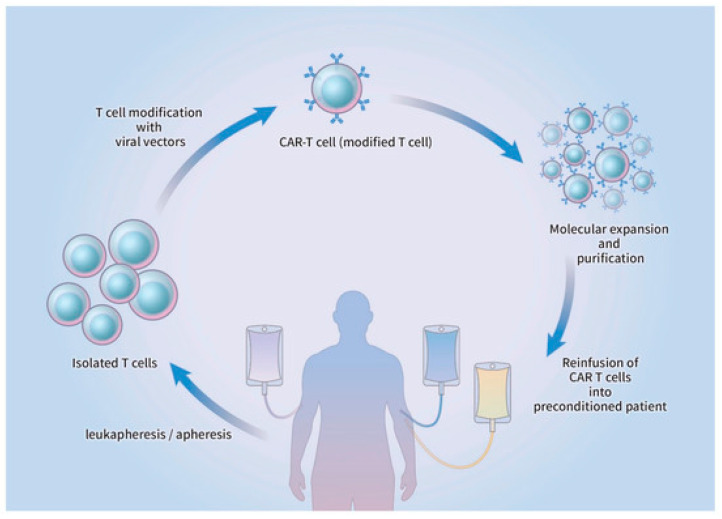
A graphic representation of chimeric antigen receptor (CAR)-T cell production. After being harvested from the peripheral circulation, leukapheresis and subsequent apheresis are used to isolate T cells. The prepared T cells undergo transduction by viral or non-viral vectors and are genetically altered to express chimeric antigen receptors. Before reinfusion, CAR-T cells are subjected to ex vivo expansion and purification and are reinfused into the lymphodepleted patient [25].

Activation of the CAR-T cells occurs through the interaction between the infused CAR and the target cell, which triggers a cytokine cascade and target cell lysis (Figure 2). Since the physical characteristics and molecular composition of each CAR are known to influence the efficacy of CAR-T cells, genetic engineering and molecular manipulation of CARs are still tentative, and further research is warranted. However, CAR-T cell therapy has displayed satisfactory outcomes in hematologic cancers, where complete response was seen in approximately 50% of the patients diagnosed with acute lymphoblastic leukemia (ALL), chronic lymphocytic leukemia, and lymphoma [42,43].

This treatment modality has not yet been proven to have the same efficacy in solid tumors shown in hematologic malignancies [44,45]. Only a small proportion of cancer-specific antigens is available in the clinical setting of solid neoplasms; therefore, CAR-T cell therapy is less likely to be successful against tumor cells. Identifying new targets and biomarkers is a priority for ongoing and future CAR-T immunotherapy treatments for EC patients.

## 4. Molecular Targets for CAR-T Cells in Endometrial Immunotherapy

As stated above, CAR-T cell immunotherapy has shown promising results for hematologic malignancies, but further studies and development are needed for gynecological tumors. Several potential molecular targets for immunotherapy could also represent potential targets for CAR-T cell treatment. This necessity is especially applicable in type II EC, which is non-estrogen-dependent, clinically aggressive, has a poor response to standard therapies, and has a poor prognosis [46]. A promising characteristic of the Type II variant EC is its high mutation rate, which may help pave the way for an effective CAR-T cell therapy if a suitable target is discovered.

### 4.1. Suppressive Protein Phosphatase Type II A (PP2A)

One of the potential targets for type II EC is the Aα subunit of the tumor-suppressive protein phosphatase type II A (PP2A). PPP2R1A is known to encode this subunit, and genetic alterations have been noted in up to 40% of type II endometrial tumors; type I EC and other gynecologic cancers, on the other hand, showed a mutation rate of only 0–7% [47]. Remmerie et al. showed that EC patients with a mutation in PPP2R1A had a poor prognosis due to the overactivation of the phosphoinositide 3 kinase (PI3K)/protein kinase B (Akt)/mammalian target of the rapamycin (mTOR) cascade [47]. Negative feedback of cytotoxic T-lymphocyte function in the immune system is elicited by PP2A [48]. A recent study by Cui and colleagues demonstrated that administration of a PP2A inhibitor (LB-100) with CAR-T cells enhanced the therapeutic efficacy in the clinical setting of glioblastoma. The authors concluded that LB-100 augmented the cytotoxic activity of CAR-T cells and improved tumor control and survival rates [49]. Therefore, inhibition of PP2A through CAR-T cell therapy may regulate the immune reaction against EC [6].

### 4.2. Human Epidermal Growth Factor Receptor 2

Human epidermal growth factor receptor 2 (HER-2) is another potential target. HER-2 overexpression is noted in breast and gastric cancers, and it is overexpressed in type II EC [46]; study results have associated HER-2 overexpression with a poor prognosis [50]. Although trastuzumab, a monoclonal antibody targeting HER-2, is currently used to treat HER-2-positive patients, clinical trials evaluating the treatment benefits in EC failed to show an outcome similar to that of breast cancer.

The underlying pathophysiology behind this difference is assumed to be an innate or acquired resistance to anti-HER-2 monoclonal antibodies [51]. Further investigation of antibody resistance should be performed before considering HER-2 target selectivity for CAR-T cell immunotherapy. Zhao and colleagues have suggested that the resistance may be due to overexpression of the phosphatidylinositol 3 kinase (PI3KCA) pathway, which regulates the downstream cascade of HER-2 [52]. PI3KCA gene mutations have been observed in advanced EC, especially within those overexpressing HER-2 [53,54]. Therefore, inserting a PI3KCA inhibitor into the CAR-T cell molecule can negate resistance against HER-2-targeting antibodies.

### 4.3. Androgen Receptor (AR)

The androgen receptor (AR) is overexpressed in EC, especially in metastatic EC. Similar differences in AR expression are observed in primary tumors compared to metastatic disease. EC patients with lesions that show higher expression of AR and lower estrogen receptor expression have a poor prognosis; these characteristics make these patients prospective candidates for CAR-T cell therapy. However, further studies are required to clarify the potential role of AR as a molecular target for CAR-T cell therapy [55].

## 5. Current and Ongoing Clinical Trials for CAR-T Cell Immunotherapy in Endometrial Cancer

In early 2021, Reiss et al. initiated the phase I, first-in-human (FIH) study of adenovirally transduced autologous macrophages engineered to contain an anti-HER2 chimeric antigen receptor (CAR) in subjects with HER2-overexpressing tumors (NCT04660929), a clinical trial [56]. This open-label study was carried out to evaluate the safety, tolerability, and manufacturing potential of CT-0508, which is an autologous macrophage engineered to contain an anti-HER-2 CAR. The focus is on patients diagnosed with HER-2-overexpressing solid tumors who have failed conventional treatment. The study groups are divided into two cohorts, and both groups will undergo CT-0508 administration. Group 1 will receive split doses via intravenous administration starting with 500 million cells when the study is initiated, eventually increasing to 3.0 billion cells after five days; group 2 will receive the full dose of up to 5 billion cells via intravenous administration at the beginning of the trial. The primary outcome measures include assessing the safety and tolerability of CT-0508 by evaluating the adverse events (AEs) related to the therapy over 14 months, focusing on cytokine release syndrome (CRS). This trial’s primary endpoints are analyzing the practicality of engineering anti-HER-2 CAR-macrophages. Secondary outcome measures, including estimating the objective response rate (ORR) and progression-free survival (PFS) for the study population, will be evaluated over 24 months. Data from the clinical trial demonstrated a favorable safety and tolerability profile in the first seven patients who were enrolled. The study population included patients with breast cancer, gastrointestinal malignancies, ovarian cancer, and salivary duct carcinoma. Moreover, no dose-limiting adverse events were reported. Five patients had CRS while two patients had AEs relating to disease progression. The investigators assessed treatment efficacy and found that four of the seven patients had stable disease at 8 weeks. Although EC patients were not included in the preliminary reports, the study inclusion criteria specify HER2-positive recurrent or metastatic solid tumors, making patients with refractory EC eligible; therefore, the therapeutic benefits of this specific CAR-T cell therapy for EC in the advanced or metastatic setting warrant further studies.

The First-in-Human Anti-ALPP CAR-T Cells Immunotherapy for Ovarian and Endometrial Cancer (NCT04627740) clinical trial is a single-arm, single-center, open-label study of anti-alkaline phosphatase placental (ALPP)-positive CAR-T cells [57]. The study population is patients with ALPP-positive metastatic ovarian and endometrial cancer. Before anti-ALPP CAR-T cell (TC-A101) infusion, cyclophosphamide is given at a dose of 20 mg/kg for one day, followed by 35 mg/m^2^ fludarabine, administered for three consecutive days. The primary objective of this study is to evaluate the cases of ALPP-positive subjects who experienced treatment-related AEs after the infusion of TC-A101. The secondary objectives include the determination of ORR to TC-A101 infusion over eight weeks, evaluation of PFS, and assessment of the quantity and percentage of ALPP-CAR-T cells in the circulatory system from ALPP-positive patients after six months of treatment. Preclinical murine studies have proven the therapeutic effects of TC-A101. Currently, three patients have received a low dosage of TC-A101 without serious AE. Tumor regression was observed in two patients while one patient achieved partial response. The clinical trial concluded that the initial findings of TC-A101 administration has treatment benefits in patients with ALPP-expressing malignancies.

Treatment of Relapsed and/or Chemotherapy Refractory Advanced Malignancies by CAR-T-meso (NCT02580747) is a phase I interventional clinical study. This trial analyzed the efficacy of chimeric mesothelin antigen receptor-modified T (CAR-T-meso) cells for patients with relapsed or chemotherapy refractory cancers [58]. The study is based on the rationale that placing a genetically engineered, tumor-specific CAR into autologous or donor T cells may enhance the host’s immune reaction to attack tumor cells. This study’s objective was to determine whether genetically modified lymphocyte therapy is safe and effective for subjects diagnosed with relapsed and/or chemotherapy-resistant neoplasms. This was achieved via reverse transcription polymerase chain reactions (RT-PCR), which analyzes whole blood samples from subjects treated with this immunotherapeutic modality. The study completion date was November 2018, and the status of this clinical trial has not been verified and updated in more than two years. Table 1 summarizes clinical trials that are ongoing with the continuous research of the therapeutic benefits of CAR-T cells for the treatment of EC patients in the advanced or metastatic setting.

## 6. Future Directions for CAR-T Cell Immunotherapy for Endometrial Cancer

CAR-T cell therapy may offer an alternative treatment for patients with treatment-unresponsive or advanced tumors. This immunotherapy has proven its effectiveness against hematological malignancies. However, CAR-T cell therapy is still in the development process for solid tumors and requires further advances to enhance treatment potency. In addition, the manufacturing process should increase the safety and efficacy of the CAR-T cell treatment while decreasing possible AE. This can be achieved by formulating a better T cell subclass and revitalizing the host immune response by boosting immune antibody and cytokine production [59].

Several studies have focused on redesigning the CAR molecules to decrease toxicity. Preclinical studies have established that engineering a CAR with decreased scFv affinity can reduce immune-mediated toxicity in normal tissue and organs [60,61]. The tumor specificity of the CAR-T cell can be improved by using a Notch receptor. The Notch receptor-connected scFv is separated after antigen binding, which enables the intracellular zone to express a second tumor antigen [62]. Other options being explored are split CARs, where the CAR domains are linked only when a small molecule with dimerizing activity is present [63]. Other options to decrease AEs might be to manufacture switchable CARs in which the CAR cassette specifically interacts with a neoepitope, which induces activation of T cells after antibody–antigen binding [64].

Although the therapeutic benefits of CAR-T cells are promising, these entities have a notoriously short life span in blood circulation, which limits their therapeutic efficacy. CAR-T cell hyporesponsiveness or exhaustion is due to the toxic background of the TME along with a decrease in cytokine production and an increase in inhibitory surface receptors. Specific and alternative methods are being explored to mitigate this inhibition and improve antitumor efficacy. Certain CAR molecules express cytokines such as IL-12, IL-15, IL-18, and IL-21 that play a significant role in T cell persistence and proliferation.

After interacting with a tumor-specific antigen, these cytokines can mitigate the immunosuppressive TME while augmenting the host immune response by redirecting the CAR-specific T cell to destroy solid tumors [33]. Moreover, a recent study demonstrated antigen-specific lysis of solid tumor cell lines with CAR-T cell administration, which supports the clinical rationale for the potential usage of this immunotherapeutic modality for the treatment of solid neoplasms [65]. Laboratory analyses have shown that the Nr4a family of nuclear receptor transcription factors initiates the nuclear factor of activated T cells (NFAT), which can induce CAR-T cell hyporesponsive states [66]. Murine preclinical studies concluded that CAR-T cells not expressing these molecules can circumvent CAR-T cell exhaustion and induce tumor reduction [67].

The development of a non-MHC-restricted, universal CAR-T cell platform, where mass production of infusion cells and individually tailored T cells is possible, could open new opportunities in treating and managing EC. The development of this proposed commercial CAR-T is likely to become a possibility as research and industrial facilities increase in size and numbers while “off-the-shelf” production becomes efficient through automation and improved manufacturing capabilities [68].

## 7. Adverse Events in CAR-T Cell Therapy

Although CAR-T cell immunotherapy has shown therapeutic potential in various studies and clinical trials, most treatment regimens were accompanied by toxicity and immune-related AEs (IRAEs). Immune-related toxicity includes CRS and tumor lysis syndrome (TLS), along with neurological side effects [69]. Moreover, anaphylaxis, graft-versus-host disease (GVHD), and “on-target/off-tumor toxicities” have been reported during treatment by CAR-T cell infusion [70].

### 7.1. Cytokine Release Syndrome

CRS is considered the most severe adverse event arising from CAR-T cell therapy. CRS is a systemic inflammation caused by elevated cytokine levels associated with T cell expansion [71]. The spectrum of severity ranges from mild symptoms to multi-organ failure. The severe form of CRS may develop into fulminant hemophagocytic lymphohistiocytosis, whose symptoms include macrophage and lymphocyte hyperactivation, upregulation of cytokines with infiltration of lymphohistiocytic cells, and immune-related multi-organ dysfunction [72]. Typical clinical features of CRS include fever, nausea, anorexia, myalgia, hypoxia, encephalopathy, and hypotension [73].

The majority of mild to moderate CRS is treatable with minimal intervention. However, CRS can escalate rapidly with the involvement of transient organ dysfunction. Severe CRS progresses into a high fever, tachycardia, and hypotension, which often results in heart failure; other clinical manifestations involving the nervous system, such as seizures and obtundation, have been associated with this adverse event [71]. Study results indicate that high levels of IL-6, which causes vasodilation, hypoperfusion, hypotension, and acute kidney injury (AKI), are closely associated with severe CRS [74]. To this end, using the anti-IL-6 receptor antibody tocilizumab results in rapid resolution of severe CRS without CAR-T cell depletion or attenuation of therapeutic benefit; it has been incorporated into the management of CRS [75].

The definitions and onset of CRS are not uniformly defined, and disease progression is hard to monitor. Hospitals and treatment centers have developed individual CRS grading scales, which make it challenging to compare symptom severity and treatment outcomes. Based on the clinical data and experience from the University of Pennsylvania, the Penn grading scale (Table 2) was developed for a more uniform grading system and an effective management algorithm [76].

### 7.2. Tumor Lysis Syndrome

The underlying therapeutic mechanism of CAR-T cells is the lysis of tumor cells. Consequently, TLS is caused by increased serum lactate dehydrogenase and uric acid levels, usually occurring approximately three weeks after CAR-T cell administration [77]. Patient monitoring protocols for the management of TLS should include serum potassium, calcium creatinine, and uric acid levels. TLS is associated with hyperuricemia, hyperkalemia, and hyperphosphatemia, along with other adverse effects. The objective of patient monitoring is the prevention of TLS, which can lead to AKI. Management of TLS includes fluid hydration, prophylactic allopurinol, and hemodialysis for critical situations. However, further research is needed to understand the mechanism of severe TLS [78].

### 7.3. Neurological Toxicities

Neurological AEs include confusion, delirium, myoclonus, seizures, obtundation, and expressive aphasia. Such symptoms seem related to CD 19-specific CAR-T cells; however, other antigen-specific treatments can elicit such events. Neurological toxicities due to CAR-T cell infusion had no direct correlation with CRS, were self-limiting, and had no long-term deleterious effects [79]. The overall objective for the management of neurological AEs is reducing the severity of the inflammatory response, which could be attained by the administration of the IL-6 antagonist Siltuximab, which inhibits continuous IL-6 translocation across the blood–brain barrier [80]. Studies have shown that dexamethasone or corticosteroids may help reduce neurological toxicities [81]. The prophylactic use of antiepileptic agents can be considered for the prevention and management of severe neurological dysfunction such as seizures [81]. Although there are no standard imaging studies to diagnose this clinical manifestation, cerebrospinal fluid (CSF) analysis has shown the presence of CAR-T cells [82]. In-depth studies are necessary to understand the molecular pathogenesis of this disease entity, associated risk factors, and the optimal CAR-T cell infusion dosage for the prevention of the aforementioned events.

### 7.4. “On-Target/Off-Tumor” Toxicity

“On-target/off-tumor” toxicity results from the direct engagement of normal tissues that share the expression of the target antigen. Taking the potency of activated T cells into consideration, the toxicity affecting non-cancerous tissues with low antigen levels is highly damaging. Early studies conducted by Lamers et al. reported the occurrence of cholestasis in patients with renal cell carcinoma after the administration of CAR-T cells specific for carbonic anhydrase IX (CaIX) [70].

The severity of this adverse event has been confirmed to be associated with dosage amount. Bonifant et al. reported a fatal case of a patient who had received a high-dose infusion of HER-2-specific CAR-T cells. This regimen caused respiratory distress and multi-organ failure [75]. Moreover, a study based on an HER-2/neu-specific CAR-T cell regimen has confirmed that a high infusion dosage can potentially cause this specific toxicity; lower dosages of CAR-T cells without chemotherapy preconditioning are considered safe [83]. Further studies are needed to regulate the threshold for this adverse event and to define the optimal dosage for the safe application of CAR-T cell immunotherapy.

### 7.5. Anaphylaxis and Graft-versus-Host Disease (GVHD)

Anaphylactic reactions and GVHD are other examples of toxicities reported in trials involving CAR-T cell therapy. GVHD is the result of alloreactivity related to the administration of non-host CAR-T cells and can be overcome by endogenous TCR silencing and CAR-transduced viral-specific cells [84,85,86]. Murine domains used in CAR molecules have resulted in anaphylactic reactions [75]. Research and clinical studies are underway to humanize the molecular domains [83]. Modifications within the molecular structure of the allogeneic CAR T-cells could mitigate the severity of GVHD, which include the utilization of genomic editing such as Zinc finger nucleases (ZFN), transcription activator-like effector nucleases (TALEN), and clustered regularly interspaced short palindromic repeats/CRISPR associated protein 9 (CRISPR/Cas9). These genetic tools can be applied in knocking-out TCR and diminishing the adverse events of GVHD [87]. Moreover, research is ongoing to alleviate allorejection; chemo-resistant CAR-T cells are being repeatedly tested through several rounds of administration to allow more profound or prolonged lymphopenia [85,88]. Close monitoring of the patients and early recognition leading to optimal management of anaphylaxis are critically important for patients undergoing CAR-T cell immunotherapy. A safe CAR-T cell immunotherapy protocol could be possible with future research and large-scale clinical trials based on novel biomarkers, early intervention, and effective countermeasures for these AE.

## 8. Conclusions

After showing promising therapeutic benefit in various EC molecular subtypes, immune checkpoint blockade was granted accelerated approval by the FDA for the treatment of EC in the advanced and recurrent settings. These results emphasize the potential benefits of immunotherapy in the management of advanced EC. Despite technological breakthroughs and advances in immunotherapy, the development of CAR-T cell technology in EC is still in its initial phases and the treatment outcomes are inadequate to be considered as standard therapy. Like other solid tumors, EC faces challenges from using engineered T cells to treat the disease. This treatment inevitably faces challenges, such as AE, safety concerns, the selection of a specific target antigen, and short therapeutic duration [65]. Further research is warranted to develop strategies to manage these limitations while increasing the therapeutic benefit of CAR-T cells in the clinical setting of EC. The goal is to achieve results comparable to those achieved for hematologic malignancies. In regard to the ethical aspects of alternative modalities, there is no clear scientific rationale or clinical evidence for testing CAR-T cells as a replacement for ICI immunotherapy. Nevertheless, with ongoing clinical trials and developments to overcome these obstacles, CAR-T cell immunotherapy may play a role in treating patients experiencing recurrence after ICI treatment in the future.

## Figures and Tables

**Table 1:** Ongoing studies regarding CAR-T cell therapy against endometrial cancer.

Study Title	Clinical Phase	Identifier	Conditions	Primary Endpoints
First-in-human (FIH) study of adenovirally transduced autologous macrophages engineered to contain an anti-HER2 chimeric antigen receptor (CAR) in subjects with HER2 overexpressing tumors	Phase I	NCT04660929	HER-2 positive Adenocarcinoma HER-2 positive Solid Tumors HER-2 Protein Overexpression HER-2 Gene Amplification	Assessment of the safety and tolerability of CT-0508 Frequency and severity of AEs Assessment of the feasibility of manufacturing CT-0508
First-in-human anti-ALPP CAR-T cell immunotherapy for ovarian and endometrial cancer	Phase I/II	NCT04627740	Endometrial Cancer Ovarian Cancer	Number of ALPP-positive participants with treatment-related AEs after infusion with anti-ALPP CAR-T cells
Treatment of Relapsed and/or Chemotherapy Refractory Advanced Malignancies by CART-meso	Phase I	NCT02580747	Endometrial Cancer Ovarian Tumor Malignant Mesothelioma Pancreatic Cancer Triple Negative Breast Cancer Other Mesothelin Positive Tumors	Safety and feasibility of CAR-T meso cells Occurrence of AE

CAR: chimeric antigen receptor, HER-2: human epidermal growth factor receptor 2, AE: adverse events, ALPP: alkaline phosphatase placental, meso: mesothelin.

**Table 2:** Penn grading scale for CRS [76].

**Grade**	**Management**
1	Mild: Treated with supportive care such as antipyretic agents, antiemetic agents
2	Moderate: Requiring IV therapies or parenteral nutrition; some signs of organ dysfunction (i.e., grade 2 Cr or grade 3 LFTs) related to CRS and not attributable to any other condition; hospitalization for management of CRS-related symptoms, including fevers with associated neutropenia
3	More severe: Hospitalization is required for management of symptoms related to organ dysfunction, including grade 4 LFTs or grade 3 Cr associated with CRS and not attributable to any other conditions; this excludes management of fever or myalgias but includes hypotension treated with IV fluids or low-dose vasopressors, coagulopathy requiring FFP or cryoprecipitate, and hypoxia requiring supplemental O_2_ (nasal cannula O_2_, high-flow O_2_, CPAP, or BiPAP); patients admitted for management of suspected infection owing to fever and/or neutropenia might have grade 2 CRS
4	Life-threatening complications such as hypotension requiring high-dose vasopressors, hypoxia requiring mechanical ventilation

BiPAP, bilevel positive airway pressure; CPAP, continuous positive airway pressure; Cr, creatinine; CRS, cytokine release syndrome; FFP, fresh frozen plasma; IV, intravenous; LFT, liver function test.

## Data Availability

No new data were created or analyzed in this study.
